# Chronic AMPK Activation Reduces the Expression and Alters Distribution of Synaptic Proteins in Neuronal SH-SY5Y Cells

**DOI:** 10.3390/cells11152354

**Published:** 2022-07-31

**Authors:** Alex J. T. Yang, Ahmad Mohammad, Evangelia Tsiani, Aleksandar Necakov, Rebecca E. K. MacPherson

**Affiliations:** 1 Department of Health Sciences, Brock University, St. Catharines, ON L2S 3A1, Canada; ay14dx@brocku.ca (A.J.T.Y.); am17jq@brocku.ca (A.M.); etsiani@brocku.ca (E.T.); 2 Department of Biological Sciences, Brock University, St. Catharines, ON L2S 3A1, Canada; anecakov@brocku.ca; 3 Centre for Neuroscience, Brock University, St. Catharines, ON L2S 3A1, Canada

**Keywords:** AMPK, mTORC1, neuronal health, post-synaptic density, Homer-1

## Abstract

Neuronal growth and synaptic function are dependent on precise protein production and turnover at the synapse. AMPK-activated protein kinase (AMPK) represents a metabolic node involved in energy sensing and in regulating synaptic protein homeostasis. However, there is ambiguity surrounding the role of AMPK in regulating neuronal growth and health. This study examined the effect of chronic AMPK activation on markers of synaptic function and growth. Retinoic-acid-differentiated SH-SY5Y human neuroblastoma cells were treated with A-769662 (100 nM) or Compound C (30 nM) for 1, 3, or 5 days before AMPK, mTORC1, and markers for synapse function were examined. Cell morphology, neuronal marker content, and location were quantified after 5 days of treatment. AMPK phosphorylation was maintained throughout all 5 days of treatment with A-769662 and resulted in chronic mTORC1 inhibition. Lower total, soma, and neuritic neuronal marker contents were observed following 5 d of AMPK activation. Neurite protein abundance and distribution was lower following 5 days of A-769662 treatment. Our data suggest that chronic AMPK activation impacts synaptic protein content and reduces neurite protein abundance and distribution. These results highlight a distinct role that metabolism plays on markers of synapse health and function.

## 1. Introduction

Alzheimer’s disease (AD) is a neurodegenerative disease defined by amyloid-β plaque deposits (Aβ) and Tau-tangles (Tau) that physically damage neuronal connections, resulting in brain atrophy. These micro- and macroscopic changes are preceded by the disruption of synaptic connections resulting in mild to severe cognitive impairments [1,2]. The development of sporadic AD is the result of progressive synaptic impairments that have been linked to metabolic risk factors such as insulin resistance (IR), type 2 diabetes mellitus (T2DM), and obesity rather than predominantly genetic components [3,4]. Recent studies point to convergent mechanisms (neuroinflammation, mitochondrial impairments, insulin resistance) and impairments in energy homeostasis leading to increased synaptic damage [5,6,7]. These similarities in etiology have prompted researchers to consider whether treatments targeting metabolism provide therapeutic benefits in preventing these observed synaptic impairments. Particular attention has been given to a key regulator of cellular homeostasis, AMP-activated protein kinase (AMPK), which is a main target in the treatment of T2DM [8,9,10,11].

AMPK is a serine/threonine kinase that is activated by AMP and plays a major role in regulating metabolic homeostasis [12,13]. By being sensitive to AMP concentrations, AMPK is a sensor of cellular energy and is activated under conditions that cause increases in the AMP:ATP ratio [14]. Due to the ability to sense energetic stress, AMPK signaling plays a central role in promoting catabolic processes that act to increase ATP levels: processes such as increased glucose uptake via greater GLUT4 translocation [15], increased fatty acid oxidation via increased CPT1 activity [16,17], improving mitochondrial function by promoting mitochondrial proliferation via PGC1-α, and the recycling of damaged mitochondria via mitophagy in skeletal muscle, liver, adipose, as well as brain tissue [18]. Previous work, in rodent models of IR, T2DM, and AD-like pathology, have also demonstrated that AMPK activation improves spatial memory [9,19,20], reduces neuroinflammation [21], and improves brain mitochondrial function [18,19].

AMPK activation also restricts anabolic processes such as cellular proliferation/growth and protein synthesis in an effort to preserve cellular ATP content through the inhibition of a major downstream target of AMPK, the mechanistic target of the rapamycin complex (mTORC1) [12]. mTORC1 is a cellular proliferation and growth complex that is similarly involved in balancing energy homeostasis. However, unlike AMPK, mTORC1 promotes cellular proliferation, protein synthesis, and lipid synthesis through activation of ribosomal S6 protein kinases and sterol regulatory element-binding proteins, respectively [20,22]. The balance between AMPK and mTORC1 activation is imperative for maintaining adaptability under energetic challenges. Importantly, AMPK directly phosphorylates the S792 site of raptor, the major complexation subunit of mTORC1, which results in the inhibition of mTORC1 activity [23].

Considering the importance of metabolic homeostasis in protein synthesis and cellular growth, the AMPK-mTORC1 signaling cascade has gained attention in some areas of AD research [12,24]. AMPK has been shown to play a significant role in synapse development and function through mTORC1 [25,26]. In the brain, activation of mTORC1 induces production of growth markers and neurotrophic factors, including Homer-1, and brain-derived neurotrophic factor (BDNF) secretion, all important for proper synapse function, and all of which are downregulated in AD [27,28,29,30]. Activation of AMPK is expected to inhibit mTORC1 and downregulate synaptic markers, raising the important question towards exactly what the role of metabolism and extent of chronic AMPK activation is on maintaining healthy cellular processes. For example, acute AMPK phosphorylation/activation may be beneficial in restoring metabolic homeostasis in the brain [31]; however, its activation has also been linked to reduced cell size, neurite length, and neuronal polarization [25]. Furthermore, there is ambiguity surrounding the role of AMPK in neurogenesis, synapse plasticity/growth, and the increased phosphorylation of AMPKs that is observed in post mortem AD brains [32,33,34]. As such, a number of questions remain regarding the role that AMPK has on synaptic health and function [35]. The purpose of this study was to examine the effect of chronic AMPK activation and inhibition on markers of synapse growth and synaptic strength. We hypothesized that prolonged (>24 h) AMPK phosphorylation/activation would lead to reductions in the content of synaptic machinery, their content at the synapse, as well as decreased synaptic growth.

## 2. Materials and Methods

### 2.1. Cell Line and Treatments

SH-SY5Y human neuroblastoma cells were cultured using high glucose Dulbecco’s Modified Eagles Media (DMEM Sigma D6429), containing 10% or 1% (*v*/*v*) FBS (Sigma F1051, St Louis, MO, USA; for seeding and differentiation respectively), 5% non-essential amino acid (Sigma M7145, St Louis, MO, USA), and 1% Penicillin/Streptomycin (Sigma P4333, St Louis, MO, USA). Differentiation was accomplished with retinoic acid (1 μg/mL) for 5 days. Differentiated cells were assigned to one of three groups and treated: (1) vehicle control; (2) A-769662 (100 nM; AMPK agonist); and (3) Compound C (30 nM; AMPK inhibitor). These concentrations are lower than any doses previously reported in the literature with this cell line [9,10,11,31,36,37,38,39,40]. Cells were treated for 1, 3, and 5 days, followed by whole cell lysate preparation and assessment of specific protein content and phosphorylation status via Western blotting (WB). Media with fresh drug aliquots were changed daily. Use of this cell line was approved by the Research Ethics Board at Brock University (#17-397). Cell viability measurements were conducted using 0.4% Trypan blue dye [41]. The results represent n = 3–6 independent experiments with each condition having 3 technical replicates. 

### 2.2. Western Blotting 

Cells were lysed in 200 μL cell lysis buffer (NP40 Cell Lysis Buffer; Life Technologies; CAT# FNN0021; Carlsbad, CA, USA) supplemented with 34 μL phenylmethylsulfonyl fluoride and 50 μL protease inhibitor cocktail (Sigma; CAT# 7626-5G, CAT# P274-1BIL; St Louis, MO, USA). Cells lysates were then sonicated (Fischer Scientific Sonic Dismembator 100). A bicinchoninic acid assay was performed to determine protein content of the lysates. Samples were prepared (1 μg/μL) and equal amounts of protein were then electrophoretically separated on 10% SDS-PAGE gels and transferred to nitrocellulose membranes (GE Life Science Ca. 10600002, 0.45 μm). After being cut to analyze multiple proteins/loading controls per gel, membranes were blocked for 90 min at room temperature in 5% non-fat dry milk-TBST (tris-buffered saline/0.1% tween 20). Membranes were then incubated in primary antibody diluted 1:1000 in 5% BSA (Bovine Serum Albumin)-TBST overnight at 4 °C with gentle agitation. The following day, membranes were incubated for 1 h at room temperature with the appropriate secondary antibodies (1:2000; Donkey anti-rabbit IgG (H + L), #711-035-152, Goat anti-mouse IgG (H + L), #115-035-003 Jackson Immunoresearch) in 1% BSA-TBST. Membranes were rinsed 3 × 5 min in TBST and proteins visualized by Western Lightning Plus-ECL (Perkinelmer NEL103E001EA, Waltham, MA, USA) using a ChemiDoc Imaging System (Bio-Rad, Hercules, CA, USA). Band densitometry was quantified using Alpha Innotech software (Santa Clara, CA, USA). A constituently expressed protein (β-actin/vinculin) was measured and analyzed for each membrane to ensure equal loading (<10% variability across the membrane). Total and phosphorylated AMPK levels were measured using specific antibodies (Cell Signaling, Danvers, MA, USA; #2531; Cell Signaling #2535—recognizes phosphorylated Threonine 172). Total raptor (Cell Signalling #2280) and phosphorylated raptor (S792, Cell Signalling #2083) levels were also measured alongside. Synaptophysin (Cell Signalling #5461), Bassoon (Pre-synaptic marker, SC-58509), Homer-1 (post-synaptic marker, SC-136358), and BDNF (Santa Cruz SC-65514) were measured as synaptic markers. Loading controls were conducted using vinculin (Millipore-Sigma, St Louis, MO, USA, 05-386) and β-actin (Abcam ab8227, Cambridge, UK).

### 2.3. Immunofluorescence

SH-SY5Y cells were seeded, differentiated, and grown on MatTek glass bottom culture dishes until they reached 75% confluency (35 mm Dish, No. 1.5 Coverslip, 10 mm Glass Diameter, Collagen Coated). Cells were then fixed using 4% paraformaldehyde and permeabilized using 20% triton X-100 before being incubated in buffer containing the appropriate primary antibodies followed by the Alexa Fluor secondary antibodies (abcam; ab150077). Bassoon and Homer-1 (as pre- and post-synaptic markers) and synaptophysin were imaged. Images were captured using a Biotek Cytation5 cell imaging reader. Immunofluorescent (IF) images were captured in a 5 × 5 image grid (10×) where images were analyzed using DAPI (abcam; ab228549) and phalloidin-actin (abcam; ab176757 staining for 2 separate outcomes: morphological changes and protein expression changes). DAPI was used to determine cell number and a phalloidin actin stain was used to determine soma size, total cell area, and projection length. Neurite length was defined by fluorescent signals measured 7–70 μm from the nucleus defined by actin stain. Cell size was defined by total cell phalloidin-actin stain, and cell soma was defined by actin fluorescence signals measured 1–7 μm outside of nucleus (defined by DAPI). Differences in protein fluorescence between cell soma (as defined by fluorescent signals measured 1–7 μm from nucleus defined by DAPI stain) and neurites (both distance and area expressed, as defined by fluorescent intensity measured 7–70 μm from nucleus defined by actin stain) were analyzed using the Biotek Gen5 imaging software. All stitched montage images were preprocessed for background flattening (rolling ball diameter 628 um, 210 pixels) on all channels before cellular analysis.

### 2.4. Statistical Analysis

Differences in protein content/phosphorylation status, cell size, neurite length, and fluorescence levels were determined using one-way ANOVA followed by a Tukey’s post hoc test. A value of *p* < 0.05 was considered significant. Western blot data are reported as mean ± SEM with results consisting of n = 3–6 independent experiments with 3 technical replicates per condition. IF results are presented as an average of the number of cells analyzed across three technical replicates per condition (1119–1903 cells). Brown–Forsythe homogeneity tests and Bartlett’s test were used to assess normality.

## 3. Results

### 3.1. Compound C and A-769662 SH-SY5Y Dose Response

**Figure 1:**
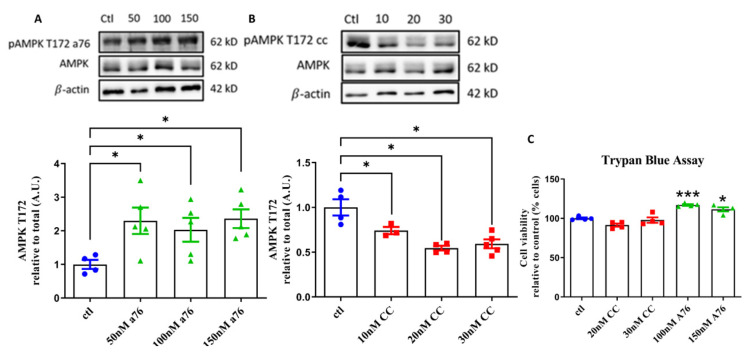
Dose response of Compound C (CC) and A-769662 (A76) on AMPK phosphorylation and cell viability. Western blot analysis for phosphorylated AMPK T172 in SH-SY5Y neuroblastoma cells following 24 h treatments of AMPK activator A-769662 ((**A**) 50 nM, 100 nM, 150 nM; green triangles) and inhibitor Compound C ((**B**) 10 nM, 20 nM, 30 nM; red squares). Control is shown as Blue circles. Representative Western blots provided above figures. Cell viability as assessed by Trypan blue exclusion assay following 7 days of treatments ((**C**) 20 nM, 30 nM, 100 nM, 150 nM). Results represent 3–5 independent experiments. Data are presented as mean ± SEM. * Indicates significant (*p* < 0.05) difference from ctl, *** Indicates significant (*p* < 0.0005) difference from ctl as determined using a one-way ANOVA followed by Tukey’s post hoc analysis.

To assess the validity of CC and A76 in modulating AMPK phosphorylation status in RA differentiation of SH-SY5Y cells, multiple, previously reported doses of both compounds [9,10,11,31,36,37,38,39] were examined over 24 h before AMPK T172 phosphorylation status was assessed via WB (Figure 1A). CC inhibits AMPK activation by acting as an AMP mimetic, binding to the γ-subunit and preventing AMP from promoting T172 phosphorylation, whereas A76 allosterically binds to the β-subunit to prevent the desphophorylation of T172 [42]. Cells were treated with either 50 nM, 100 nM, or 150 nM A76 (Figure 1A) and 10 nM, 20 nM, or 30 nM CC (Figure 1B). For both CC and A76, all three doses chosen for each drug were effective at modulating AMPK phosphorylation status at 24 h (Figure 1), with no loss in cell viability (as determined via Trypan blue assay, 7 day treatment; Figure 1C); 30 nM CC and 100 nM A76 were used for subsequent experiments.

Interestingly, while neither A76 dose decreased cell viability following 7 days of treatment, both doses caused an increase in viability of which we are unsure as to why. A76 has been reported to decrease cell viability; however, these studies report usage at much greater concentrations than were used in this study, and were not performed in the SH-SY5Y cell line [43,44]. 

### 3.2. Chronic Effects of Compound C and A76 on AMPK and Raptor

**Figure 2:**
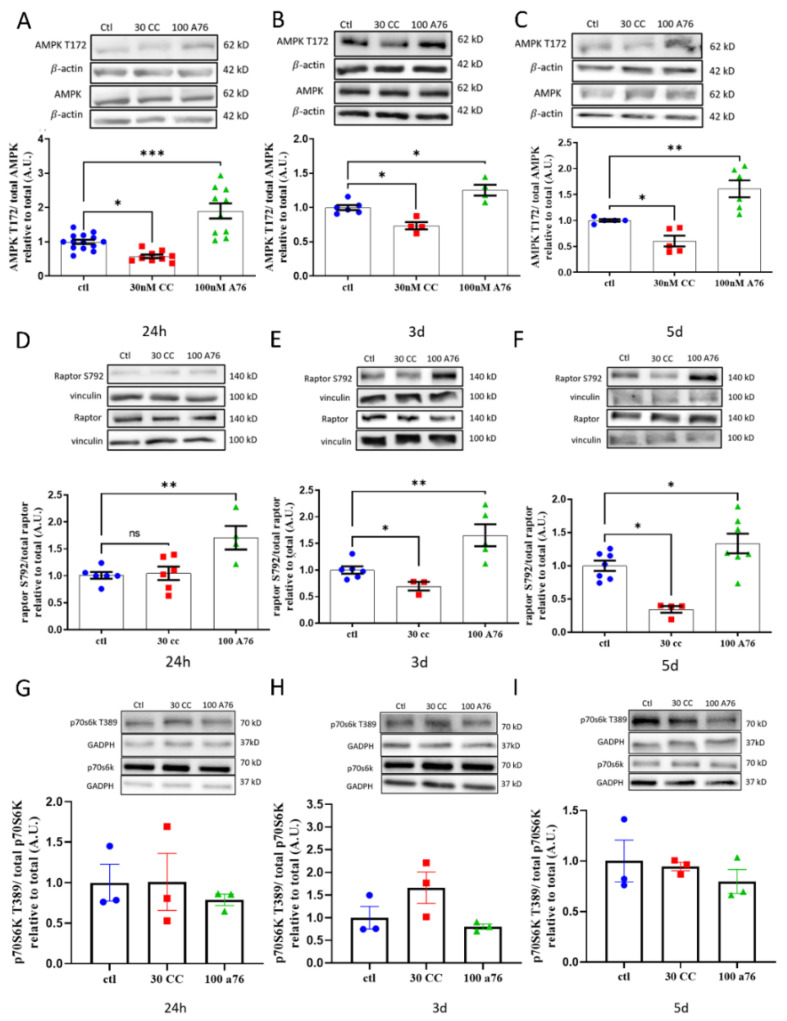
Chronic effects of Compound C (CC; Red) and A-769662 (A76; Green) on AMPK, raptor, and p70S6K. Western blot analysis for phosphorylated AMPK T172 (**A**–**C**), Raptor S792 (**D**–**F**), and p-p70S6K T389 (**G**–**I**) in SH-SY5Y neuroblastoma cells following 24 h, 3 d, and 5 d treatments of AMPK activator A-769662 (100 nM) and inhibitor Compound C (30 nM). Control is shown as Blue circles. Representative Western blots provided above figures. Results represent independent experiments (N = 3–8). Data are presented as mean ± SEM. * Indicates significant (*p* < 0.05) difference from ctl, ** Indicates significant (*p* < 0.005) difference from ctl, *** Indicates significant (*p* < 0.0005) difference from ctl as determined using a one-way ANOVA followed by Tukey’s post hoc analysis.

SH-SY5Y cells treated with 100 nM A76 for 1, 3, or 5 days showed significant increases in AMPK T172 phosphorylation compared to control (Figure 2A–C). Treatment of the cells with 30 nM CC for 1, 3, or 5 days resulted in significant reduction in AMPK T172 phosphorylation compared to control (Figure 2A–C). Given the role of AMPK in regulating protein/cell synthesis, we then examined the influence of AMPK phosphorylation on mTORC1 formation. AMPK phosphorylates raptor on S792, preventing mTORC1 complex formation [23,45,46]. Treatment of cells with 100 nM A76 for 1, 3, or 5 days resulted in higher raptor S792 phosphorylation compared to control (Figure 2D–F), whereas only treatment with 30 nM CC for 3 and 5 days resulted in lower raptor S792 phosphorylation compared to control (Figure 2E,F). To confirm the effect of AMPK activation on the modulation of the mTORC1 activity, the downstream marker phosphorylation of p70s6k was examined. p70s6k is a well-described marker directly targeted by mTORC1, whose increase in phosphorylation is indicative of increased mTORC1 activity [22]. No change in p70s6k phosphorylation was observed at any timepoint with either treatment despite changes in raptor phosphorylation status (Figure 2G–I). Overall, our treatments with A76 and CC altered AMPK phosphorylation status as well as the phosphorylation status of a marker directly associated with mTORC1 activity (raptor).

### 3.3. Synaptic Protein Content

We next examined if chronic AMPK activation or inhibition had an effect on synaptic protein content. Synaptophysin, Homer-1, and BDNF are key markers of neuronal health and plasticity that are decreased in AD and can be regulated by the AMPK signaling cascade [26,27,47,48,49,50,51]. 

**Figure 3:**
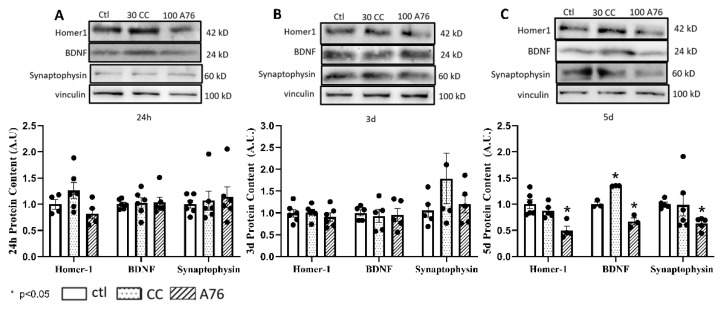
Chronic effects of Compound C (CC; dotted bars) and A-769662 (A76; hatched bars) on regulating cellular synaptic protein content. Western blot analysis for Homer-1, BDNF, and synaptophysin relative to control (open bars) in SH-SY5Y neuroblastoma cells following 24 h (**A**), 3 d (**B**), and 5 d (**C**) treatments of inhibitor Compound C (30 nM) and AMPK activator A-769662 (100 nM). Representative Western blots provided above. Results represent independent experiments (N = 3–6; Black Circles). Data are presented as mean ± SEM. * Indicates significant (*p* < 0.05) difference from ctl as determined using a one-way ANOVA followed by Tukey’s post hoc analysis.

No changes in synaptophysin, Homer-1, or BDNF protein levels were observed following 24 h or 3 days of treatment of SH-SY5Y cells with CC or A76 (Figure 3A,B); however, a significant reduction in all neuronal marker levels was seen following 5 days of A76 treatment (Figure 3C), while BDNF content showed significant increases in content following 5 days of CC treatment (Figure 3C). 

### 3.4. Cell Morphology and Synaptic Marker Content and Location

As AMPK signaling has been implicated in neuron growth and neurite polarization [25,31], we examined the effects of our treatments on neuron morphology and protein location. Following 5 days of treatment with 100 nM A76 or 30 nM CC, soma size, total cell area, and neuritic projection length were examined using IF imaging. Soma size, total cell area, and neurite projection length were determined in reference to phalloidin actin staining. Neurite projections were defined by phalloidin-actin staining and were determined to be 7–70 μm outside of the cell soma. Protein content within the 7–70 μm region was examined next. Changes in protein content were quantified by measuring total cell secondary antibody fluorescent expression. Protein expression within projections was determined by the distance of secondary antibody fluorescent expression 7–70 μm outside of cell soma, within neurite regions defined by phalloidin-actin staining. Total area of fluorescent expression was also measured within these projections and was used as a representative of protein expression/availability at a synapse. For these experiments, the presynaptic marker Bassoon was added as a better representation of presynaptic protein expression rather than the more ubiquitous BDNF that is expressed in both the neurite projections and cell body [52]. Bassoon is tightly associated with the presynaptic active zone [52,53], involved in mediating synaptic transmission [54], and whose loss has been shown to directly promote synaptic degradation [52]. Reductions in Bassoon have also been observed with AD progression [27]. 

**Figure 4:**
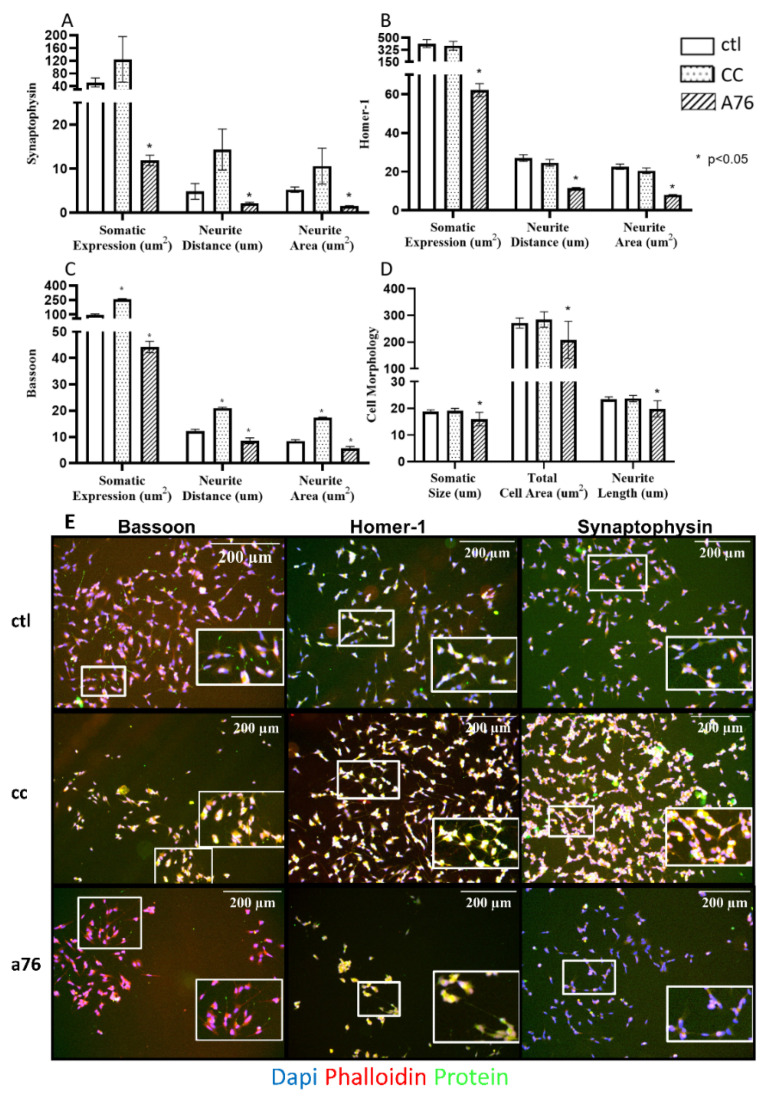
Chronic effects of Compound C (CC) and A-769662 (A76) on regulating cell morphology, protein content, and cellular location of synaptic markers. Total cell area (total protein content), projection length (distance of protein expression in projections), and projection area expression (projection protein content) for synaptophysin (**A**), Homer-1 (**B**), Bassoon (**C**), and change in neuron size (cell diameter), area (total cell size), and projection length (**D**) in SH-SY5Y cells relative to ctl following 5 d of A-769662 (100 nM) and Compound C (nM) treatment. Representative IF images shown below (**E**) for Bassoon, Homer-1, and Synaptophysin (N = 2–3, ~700–2500 cells per N) following 5 d of A-769662 (100 nM) and Compound C (30 n M) treatment. DAPI nucleus stain shown in blue, phalloidin cytoskeleton actin stain shown in red, and each protein of interest shown in green. Representative images are combinations of all 3 channels. Insets with increased magnification for each representative are shown by a bold, white border. Results represent independent experiments (n = 5–6). All stitched montage images were preprocessed for background flattening (rolling ball diameter 628 um, 210 pixels) on all channels before cellular analysis. Data are presented as mean ± SEM. * Indicates significant (*p* < 0.05) difference from ctl as determined using a one-way ANOVA followed by Tukey’s post hoc analysis.

Treatment with 30 nM CC had no significant effect on any marker of cell morphology; however, treatment of the cells with 100 nM A76 for 5 days resulted in significant reductions in soma size, total cell area, and projection length relative to controls (Figure 4D). With A76, a reduction in total cell expression of synaptophysin, Homer-1, and Bassoon (Figure 4A–C) was observed alongside a significant reduction in the fluorescent expression of synaptophysin, Homer-1, and Bassoon for both distance projected as well as area of expression within a neurite (Figure 4A–C). Following CC treatment, Bassoon showed increases in total cell expression, distance of expression with neurites, and area of expression within neurites, indicating a greater expression and potential availability of Bassoon at the synapse with CC (Figure 4D). 

## 4. Discussion

AMPK plays a large role in a number of metabolic diseases and has therefore become an important therapeutic target [13,23,55,56]. Impairments associated with metabolic factors such as IR and neuroinflammation seen with aging and in AD are known to exacerbate Aβ and Tau accumulation, leading to impaired brain function [57,58,59]. For these reasons, improving metabolic activity through AMPK has become an attractive target for neurodegenerative diseases. Indeed, in a model of Parkinson’s disease, increased AMPK phosphorylation/activation seems to rescue SH-SY5Y cells from degeneration [60]. However, unlike what is observed in Parkinson’s disease, AMPK has been shown to be hyperphosphorylated in post mortem AD brains [33,34,61]. When coupled with the large metabolic dependence that synapses require [62,63], the intricate role that AMPK signaling, and metabolism in general, plays on synaptic growth/function remains to be explored.

In this work, we investigated the effect of acute (24 h) and chronic (3–5 days) AMPK activation on markers’ neuronal growth. Our results demonstrate that chronic activation (5 d) of AMPK and mTORC1 inhibition via raptor phosphorylation negatively impacted cell morphology and reduced key markers of synaptic plasticity and neuronal growth in SH-SY5Y neuroblastoma cells. Specifically, we demonstrate that 5 days of AMPK activation reduced total content of a ubiquitous synaptic marker, synaptophysin, marker of post-synaptic stability Homer-1, and the major neurotrophic factor BDNF. Further, neurite distribution of synaptic marker content (defined by the content of a given protein within a neurite) was also reduced after 5 days of AMPK activation and was consistent with the reductions in total cell size, area, and projection length. These results were only seen with 5 days of AMPK activation, highlighting a distinct role for AMPK activity to affect synaptic markers in a time-dependent manner. 

The ability to maintain and respond to stressors is paramount to maintaining healthy brain homeostasis, especially through a major regulator such as AMPK [44,56,64]. However, the time frame of AMPK activation is crucial. While acute activation of AMPK has been demonstrated to be important in proper synaptic development [31], chronic AMPK hyperphosphorylation is observed in many metabolic diseases, including AD and Parkinson’s disease, and has been shown to play a major role in disease progression [10,21,36,61,65]. To this end, chronic AMPK dysregulation likely severely limits the ability of cells to appropriately respond to other stressors such as neuroinflammation and Aβ plaque buildup [66]. More so, chronic AMPK-induced restrictions of mTORC1 likely impact neuronal and synaptic cell growth and protein synthesis, leading to impairments in synaptic transmission and, ultimately, cognitive decline [10,11,25]. Reductions in neurite projection and area observed with chronic A76 treatments support this idea. Moreso, the influence that reductions in Bassoon have on degrading synaptic vesicles and synapses alike support its role in mediating these changes in neurites [52]. While it is unclear if the reductions in Bassoon drive the reductions in Homer-1 and synaptophysin, the impact of AMPK activation on these markers adds to this growing discussion for the role of metabolic homeostasis in the maintenance of proper synapse function. 

It is important to note that, though it has not been shown to occur specifically in SH-SY5Y neuroblastomas cells, both CC and A76 have been shown to have AMPK-independent actions [67,68]. Despite CCs known AMPK-independent activity, its use was selected to contrast our hypothesis that chronic AMPK activation would significantly impact synaptic marker function. As AMPK inhibition has been shown to occur with CC alongside its off-target effects [69], the lack of AMPK-activated reductions in neuronal markers seen with CC should still be considered valid provided the activator used is of a sufficient specificity. As such, the results shown in this study with the use of CC and A76 should still be considered of significance. This is of particular note when referencing the lack of change in the CC morphology data; as CC promotes the inhibition of AMPK through its binding to the active site of the γ-subunit as an AMP mimetic, there is no direct stimulation for the activation of mTORC1 as evidenced by the lack of change in p70s6k phosphorylation (Figure 2C). Similarly, there is no indication that CC has an off-target effect that influences mTORC1. As such, the lack of change in cell morphology in the presence of CC is not unexpected in a model where no mTORC1 stimulator was present (such as rapamycin).

Our results demonstrate an AMPK time-dependent action on synaptic markers that are linked to proper activity-dependent synapse formation, stability, and transmission [47,48,51,70,71]. The idea that chronic AMPK activation regulates synaptic health/growth is not unfounded; Amato et al. [25] showed that prolonged treatment (60 h) of cultured hippocampal neurons with AICAR directly impaired neuritic growth, size, and morphological polarization. Similarly, Potter et al. [11] demonstrated significant impairments in high-frequency stimulation (HFS) of isolated hippocampal neurons driven by metformin and 2-deoxy-D-glucose (2DG) AMPK activation. Our findings of impaired morphology as well as reductions in pre-/post-synaptic markers associated with LTP support the findings of Amato et al. [25] and Potter et al. [11]. Furthermore, Potter et al. demonstrated increased activation of mTORC1 with HFS that was blunted when treated with 2DG, suggesting that mTORC1 activation plays a significant role in synaptic function through increased S6K dendritic protein translocation. In support of this, Li et al. [26] demonstrated that over a period of 72 h of mTORC1 activation by ketamine that neuronal markers PSD95, GluR1, and synaptophysin showed significant increases in content. These same markers were reduced following treatment with a potent mTORC1 inhibitor, rapamycin. Furthermore, Li et al. [26] examined 1 h and 6 h of mTORC1 activation in PFC pyramidal neurons and found that the expression of PSD95, GluR1, and synaptophysin was only increased following 6 h of mTORC1 activation, supporting our observations that changes in synaptic protein content are time dependent. Didier et al. [31] also importantly showed that early markers of brain growth such as Arc, c-Fos, and Egrl are upregulated following 2 h of AMPK activation, highlighting a role for acute AMPK phosphorylation in early neuronal gene expression. This previous work, as well as our own results, indicate a role for AMPK in regulating synaptic protein content through mTORC1 and point to the importance of balanced activation of AMPK in synapses [34]. Our results support and highlight a role for AMPK in regulating synaptic protein content in healthy neuronal cells. However, despite abundant evidence supporting the role of mTORC1 on regulating synaptic function, there remains significant gaps in our understanding regarding the mechanisms by which these changes occur. In addition, given the importance of metabolic dysregulation on the progression of AD and synaptic impairments [72,73,74], it remains unclear if AMPK modulation will affect synaptic processes similarly as in models of metabolic stress such as IR and obesity, making examining the influence of AMPK on synaptic function and cognitive decline in models of metabolic dysregulation (IR, obesity) a necessary next step.

The results of this study provide a basis for future work examining the connection between AMPK activity and synaptic markers. Here, we utilized the SH-SY5Y cell line, as it has been used widely in experimental neurological studies, including analysis of neuronal differentiation, metabolism, and function related to neurodegenerative processes, neurotoxicity, and neuroprotection [75,76,77,78]. Further, these cells have been established for use in immunofluorescent observations of neuronal processes [79,80,81]. Finally, the SH-SY5Y cell line possesses only neurons, rather than the multiple cell types seen within primary neuronal cultures, allowing for the assessment of pharmacological changes that specifically impact neurons. This lent itself towards this study’s focus on specific over-activation of AMPK signaling on markers of synaptic function as a proof of concept. Moving forward, primary neuronal cultures may be a logical next step in assessing the role of AMPK and metabolism on synaptic function when paired with the influence of multiple neuronal cell types, including glia.

## 5. Conclusions

This study provides novel evidence for the role of chronic AMPK activation in regulating the expression and localization of markers directly related to synaptic stability and neuronal health. Importantly, this study showed significant impairments in these markers after chronic activation of AMPK (5 days), but not acutely (24 h–3 d). These results highlight a role for a timeframe of AMPK activation that plays a major role in regulating proper neuronal health. Coupled with reductions in cell morphology and synaptic machinery, our data provide further support for the role of metabolism and synapse health. As this study focused on the chronic modulation of AMPK under non-metabolically strenuous conditions, the impact of AMPK modulation should be further explored under metabolically strenuous conditions in order to further elucidate how metabolism can influence synaptic function. 

## Data Availability

The datasets used and/or analyzed during the current study are available from the corresponding author on reasonable request.
